# PCAT‐1 promotes cell growth by sponging miR‐129 via MAP3K7/NF‐κB pathway in multiple myeloma

**DOI:** 10.1111/jcmm.15035

**Published:** 2020-02-12

**Authors:** Xianjuan Shen, Shan Kong, Qian Yang, Qingqing Yin, Hui Cong, Xudong Wang, Shaoqing Ju

**Affiliations:** ^1^ Department of Laboratory Medicine Affiliated Hospital of Nantong University Nantong China; ^2^ Research Center of Clinical Medicine Affiliated Hospital of Nantong University Nantong China

**Keywords:** lncRNA, MAP3K7, miRNA, multiple myeloma, NF‐κB

## Abstract

Loss of one or some specific miRNA‐mediated regulation is closely associated with malignant progression of multiple myeloma (MM). But how these miRNAs work and what role the specific miRNA plays in this process of malignant progression remain unclear. It was found in this study that the expression of miR‐129 was decreased in both MM cell lines and newly diagnosed MM patients. Further clinicopathological statistics showed that miR‐129 was correlated with the isotype of MM patients. MiR‐129 overexpression disturbed cell proliferation, cell cycle evolution and spurred apoptosis both in vitro and in vivo. MAP3K7, a kinase able to activate NF‐κB circuit, was found to be up‐regulated in MM and contain a binding target of miR‐129. In addition, lncRNA PCAT‐1 functioned to sponge miR‐129 and thereby lowered its expression. PCAT‐1 knockdown eliminated the tumour‐promoting effect caused by miR‐129 inhibition, probably through repressing MAP3K7 and subsequent NF‐κB activation. To the best of our knowledge, this is the first study to have discovered that increased expression of PCAT‐1 could augment cell proliferation and cycle procession and inhibit apoptosis by down‐regulating miR‐129 via the MAP3K7/NF‐κB pathway in MM.

## 1 INTRODUCTION

Multiple myeloma (MM) is an incurable malignancy in the haematopoietic system involving the build‐up of malignant plasma cells (PC) in the bone marrow, hypercalcaemia, renal insufficiency and osteolytic bone disease.^1^ The disease involves multiple mechanisms associated with molecules and cells including mutation, chromosomal abnormality, epigenetic alteration and unbalance of stromal cells and PCs in the bone marrow microenvironment.^2^ Despite the advent of major advances in MM diagnosis and treatment in the past decade, the 5‐year survival rate of this malignant disease remains no more than 40%, partly due to the lack of precision therapies targeting specific oncogene involved in the pathogenesis.^3^ Hence, it is necessary to explore the cellular signals and molecular mechanisms associated with MM for the sake of developing novel therapeutic interventions.

The role of microRNAs (miRNAs) in post‐transcriptional regulation has been documented in the literature.^4^ Loss of miRNA regulation was reported to be closely associated with several tumour phenotypes in MM, including proliferation, drug resistance, apoptosis and metastasis. Besides, some miRNAs involved in the regulation of several key tumour‐related proteins (p53) or cytokines (IGF‐1, NF‐κB) could function as therapeutic targets in MM.^5^

MiR‐129 is a recently discovered tumour‐associated miRNA found to be dysregulated in cancers. Diao et al^6^ demonstrated that miR‐129 combined with TGIF2 could disturb glioma progression. In the case of glioblastoma multiforme, miR‐129 blocked the ability of cell proliferation, invasion, migration, neurosphere formation, angiogenesis and temozolomide resistance via Wnt5a by blocking the PKC/ERK/NF‐κB and JNK pathways.^7^ In prostate cancer (PCa), miR‐129 also played a tumour‐suppressive role by inhibiting cell proliferation, invasion and migration via targeting ETS1.^8^ Except for the above reports that identified miR‐129 as a tumour suppressor, miR‐129 was also documented to have oncogenic characteristics when targeting lamin A in triple‐negative breast cancer.^9^ In addition, elevated miR‐129‐induced ERβ loss in colorectal cancer (CRC) facilitated cell proliferation and migration.^10^ The contradictory characteristics of miR‐129 in different types of tumours add complexity to the research on miR‐129, and its biological function along with the underlying molecular mechanisms remains largely unknown in MM.

In the present study, we targeted miR‐129 and further explored its biological function and clinical application in MM. To explore its cellular mechanism, we artificially up‐ or down‐regulated miR‐129 in MM samples and cell lines, and found that miR‐129 expression enhancement diminished cell proliferation and cell cycle progression, and spurred apoptosis both in vitro and in vivo. Notably, we showed that MAP3K7 along with NF‐κB signal could affect cell division and apoptosis under the regulation of miR‐129. Additionally, we identified that lncRNA PCAT‐1 could potentially function as a competing endogenous RNA (ceRNA) of miR‐129 to augment MAP3K7. These observations may provide novel targeted therapies for MM.

## 2 MATERIALS AND METHODS

### 2.1 Patients

A cohort of 34 patients who were diagnosed with myeloma at their first clinical visits and admitted to the Affiliated Hospital of Nantong University (Nantong, China) between February 2015 and July 2018 were included in this study. The diagnosis, stage and risk status of MM were made in accordance with the National Comprehensive Cancer Network (NCCN) (2015 version 3 and 2017 version 3).^11^ All patients in this research were newly diagnosed without being administered with any treatment. Mononuclear cells (MNCs) were isolated from BM aspirates of MM patients by Ficoll‐Hypaque (Pharmacia) density sedimentation. CD138+ cells from MNCs were isolated with a BD FACSAria II using a phycoerythrin (PE)‐conjugated anti‐CD138 (BD Biosciences) antibody. The normal bone marrows from healthy donors were collected as controls. Following identification of all the samples (prior to initiating the treatment), the local ethics committee issued the approval of the protocols involved with requisite consent from the patients.

### 2.2 Cell culture

Human MM cell lines (U266, NCI‐H929 and RIMP 8226 cells) were cultured in RPMI‐1640 medium containing 10% foetal bovine serum (FBS) (Gibco BRL), 1% streptomycin‐penicillin and 1% glutamine in a humidified 37°C incubator with 5% CO_2_.

### 2.3 RNA extraction and real‐time PCR

Blood samples of patients and healthy controls were collected using plastic gel vacuum blood collection tubes and centrifuged at 1200 *g* for 10 minutes. The separated serum was placed in RNase‐free centrifuge tubes and stored at 80°C for use. Serum RNA was obtained using the serum RNA extraction kit (Life Technologies), while total RNA in cells was lysed within TRIzol reagent (Takara). Reverse transcription to obtain complementary DNA was conducted with the reverse transcription kit (Thermo Fisher Scientific) in accordance with the prescribed protocol. All amplified procedures were performed in ABI 7500 PCR Detection System (ABI). The primers involved in this study were as follows: PCAT‐1: F5′‐GAGAGCTGACATAGGCACCC‐3′ and R5′‐TCTCCACTGGTGTTCATGGC‐3′; GAPDH: F5′‐TGATGACATCAAGAAGGTGGTGAAG‐3′ and R5′‐TCCTTGGAGGCCATGTGGGCCAT‐3′. TaqMan miRNA assays were utilized for analysing miRNAs in adherence to prescribed procedures (RiboBio). All assays were done in triplicate. Relative quantitative method (2^−ΔΔCt^) was utilized to calculate the expression.

### 2.4 Cell transfection

LncRNA PCAT‐1 vectors, including the empty vector, PCAT‐1 overexpression vector, PCAT‐1 negative control and shRNA were synthesized and purchased from Shanghai GenePharma Co., Ltd. The vectors used for miR‐129 overexpression and knockdown were synthesized by RiboBio. Six‐well plates were used to seed 1 × 10^6^ cells per well followed by transfection with the vectors packaged by Lipofectamine 3000 Reagent (Invitrogen) in adherence to the prescribed procedures.

### 2.5 Measurement of cell proliferation

The cell proliferation ability was measured by Cell Counting Kit‐8 (Beyotime). 3000 MM cells/well were seeded in a 96‐well plate and cultured for 2 days, to which 10 μL CCK‐8 reagents were added. Following 2‐hours incubation at 37°C, absorbance at 450 nm was recorded for each well using 650 nm as reference. Each group had 5 biological replicates, and each experiment was repeated three times.

### 2.6 Cell cycle and apoptosis assay

After 48‐hours transfection, a PE Annexin V apoptosis detection kit (BD Pharmingen) was used for FACS in adherence to the prescribed procedures. Propidium iodide cell cycle detected kits of the same company were used to analyse the proportion of cells in each stage according to the prescribed procedure. All assays were conducted in triplicate.

### 2.7 Soft agar colony formation assay

Approximately 1000 cells per well diluted with RPMI‐1640 medium plus 20% FBS, along with 3% low melting point agarose solution, were seeded in 6‐well plates. Following 10‐day culture, the number of colonies defined as >50 cells/colony was counted.

### 2.8 Immunoblot analysis

Following extraction of total protein with RIPA, the lysate containing 1% PMSF was separated by sodium dodecyl sulphate‐polyacrylamide gel electrophoresis (SDS‐PAGE) and transferred to the PVDF membrane, followed by application of 5% BSA or skin milk for blocking. The appropriate primary antibody was applied at 4°C overnight followed by the secondary antibody for 2 hours. Protein bands were detected using enhanced chemiluminescence (ECL, Amersham Pharmacia). Triplicate assays were conducted.

### 2.9 RNA immunoprecipitation

The Magna RIP™ RNA‐Binding Protein Immunoprecipitation Kit (Millipore) was used in adherence to the prescribed procedures. While AGO2 antibody (Cell Signaling Technology) was utilized, the co‐precipitated RNA was verified. AGO2‐specific binding was confirmed with the use of controls for total RNAs (input controls) and IgG in simultaneous assays.

### 2.10 Luciferase reporter assay

PCAT‐1 fragment with the predicted binding site to miR‐129‐5p binding site was cloned into a psiCHECK‐2 luciferase reporter to form the reporter vector psiCHECK‐2‐PCAT‐1‐wild‐type (PCAT‐1‐wt). The PCAT‐1‐miR‐129‐5p binding site was mutated as indicated and named as psiCHECK‐2‐mutated‐type (PCAT‐1‐mut). Transfection of psiCHECK‐2‐PCAT‐1‐wt or psiCHECK‐2‐PCAT‐1‐mut was done along cotransfected with miR‐129‐5p mimics or control with Lipofectamine 3000 (the procedure has already been mentioned above). A 96‐well plate was used to seed 5000 HEK‐293T cells per well. Following 48‐hours transfection, Dual‐Luciferase Reporter Assay System (Promega) was applied by adherence to the prescribed procedures. Similar to the above procedures, the putative miR‐129‐5p sequence for binding the 3′‐UTR of MAP3K7 and MAPK1 was used for construction of vectors and transfection. Triplicate assays were conducted.

### 2.11 Xenograft construction in nude mice

Pathogen‐free conditions were applied to maintain BALB/c female nude mice (4 weeks old) with the approval from the Animal Care Committee of Nantong University where the study was performed. Approximately 3 × 10^6^ U266 cells transfected with miR‐129 mimics or NCs were injected into the nude mice subcutaneously. Tumour growth was monitored every 3 days, while the tumour volume was determined by length × width^2^ × 0.5. Euthanization was done 35 days later, and at the same time, excision of tumours was done so as to image and get tissue sections for subsequent immunohistochemistry.

### 2.12 Immunohistochemistry study

Tumour samples were paraffin‐embedded and stained to observe the relative expression and location of the target proteins using the avidin‐biotin‐peroxidase approach. The degree of tumour proliferation was evaluated by observing Ki‐67, and apoptosis was evaluated by caspase‐3 under a microscope (Olympus) at 200× or 400×.

### 2.13 Statistical analysis

Data analysis was completed using SPSS software 18.0 and GraphPad Prism 6.0. All data are expressed as the means ± SD obtained in triplicate assays. Mann‐Whiney *U* test was used to compare the differences between two groups. Kruskal‐Wallis *H* test was used for multiple comparisons between the groups. *P* < .05 was considered statistically significant.

## 3 RESULTS

### 3.1 miR‐129 is decreased significantly in MM patients and cells

**Figure 1:**
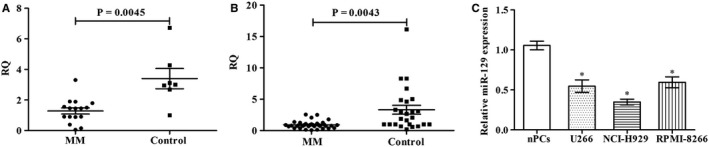
miR‐129 expression in MM samples and cells. A, Detection of miR‐129 in 16 newly diagnosed MM samples and 7 healthy donors' tissues by real‐time PCR. B, Detection of the relative expression of serum miR‐129 in patients and healthy controls. C, miR‐129 levels in three MM cell lines and nPCs cells. Minimum triplicate assays were conducted, mean ± SD, *P < .05

We compared miR‐129 expression in fresh bone marrow obtained from 16 MM patients with that from 7 healthy controls and found that miR‐129 expression was significantly lower than that in the healthy controls (Figure 1A). Additionally, similar to the bone marrow samples, the serum miR‐129 level in the 34 MM patients was lower than that in the healthy controls (Figure 1B). Serum miR‐129 expression was also associated with the clinicopathological features of the MM patients. Statistics showed that there was no significant difference in serum miR‐129 in terms of sex, age and the ISS stage in the MM patients (*P* > .05), but there was significant difference between different isotopes (*P* = .024) (Table 1). Furthermore, we also detected miR‐129 expression in MM cell lines *vs.* normal bone marrow–derived plasma cells (nPCs) and found that miR‐129 expression was lower in MM cells (Figure 1C).

**Table 1:** Correlations between the relative expression of serum miR‐129 and clinicopathological features in 34 MM patients

Clinicopathological features	Cases	miR‐129 relative expression $\left(\bar{x}\pm s\right)$	*P* value
Age (y)			
≤60	12	0.933 ± 0.709	.871
>60	22	0.910 ± 0.538	
Sex			
M	20	0.971 ± 0.561	.473
F	14	0.843 ± 0.651	
Isotype			
IgG	9	0.762 ± 0.239	.031*
IgA	5	0.443 ± 0.355	
Unclassified	20	1.105 ± 0.674	
International staging system			
Stage 1	8	0.880 ± 0.768	.435
Stage 2	12	1.010 ± 0.537	
Stage 3	14	0.862 ± 0.566	

* *P* < .05.

### 3.2 Overexpression of miR‐129 reduces cell division, arrests cell cycle and induces cell apoptosis

**Figure 2:**
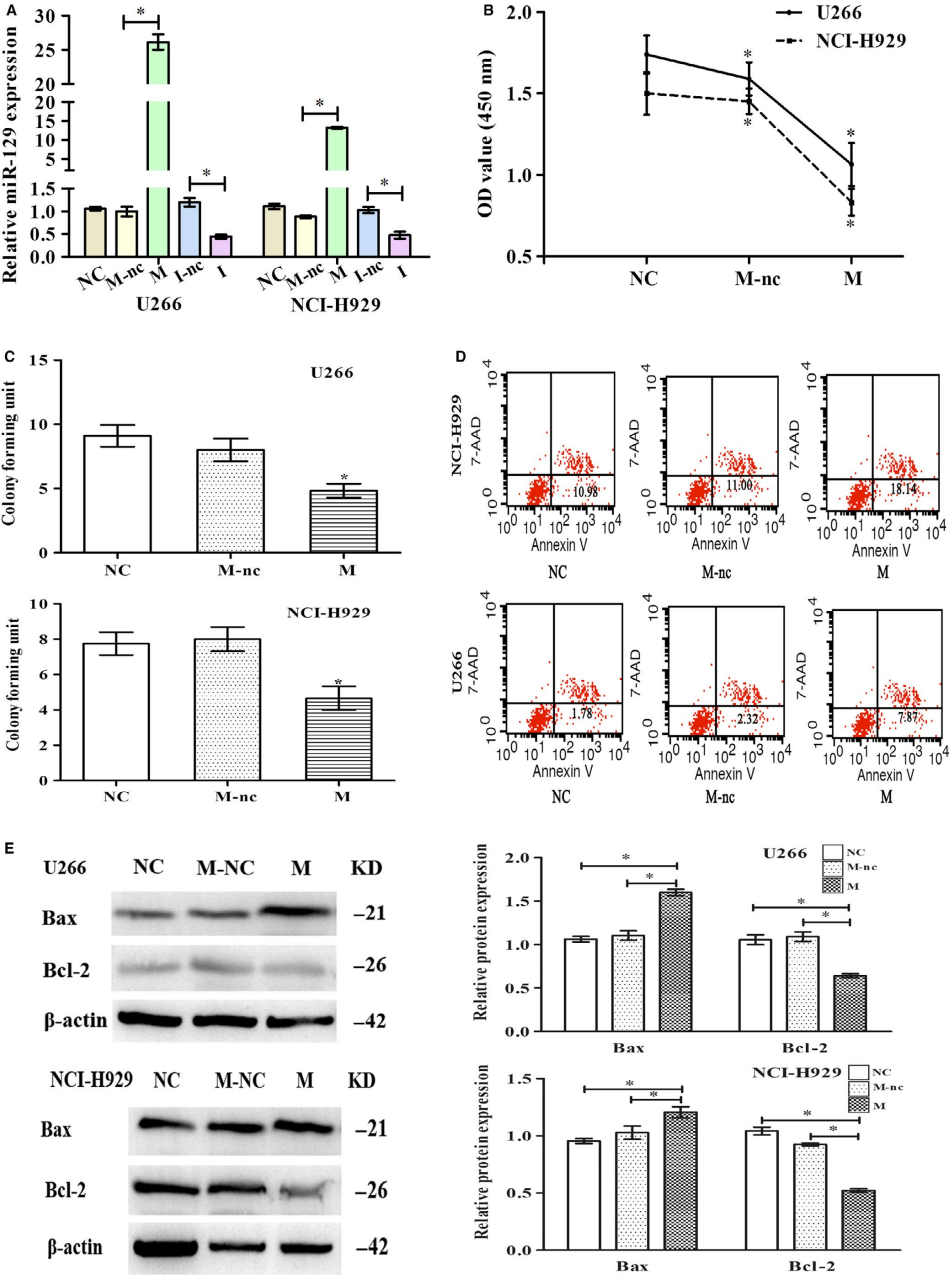
miR‐129 arrests the cell cycle, reduces the ability of cells to proliferate and induces apoptosis. Transfection of NCI‐H929 and U266 with miR‐129‐mimics (M), miR‐129‐mimics‐NC (M‐nc), miR‐129‐inhibitor (I), miR‐129‐inhibitor‐NC (I‐nc) or controls (NC). A, Detection of the relative expression of miR‐129 after transfection by real‐time PCR. B, Detection of cell proliferation by CCK‐8. Cell proliferation inhibition rates of U266 and NCI‐H929 cells in the M group were about 35% and 42%. C, The number of cloned cells as shown by soft agar colony formation assay. D, The number of apoptotic cells by flow cytometric analysis. Five independent experiments were conducted. M group owned higher apoptosis rate than the M‐nc and NC groups in U266 (*P* = .006, *P* = .007) and NCI‐H929 cells (*P* = .011, *P* = .006). E, Western blot for expression of Bcl‐2/Bax (apoptotic proteins). The results could be reproduced in 3 independent experiments, mean ± SD, *P < .05

The role of miR‐129 in MM was explored through the transfection of mimics (M), inhibitor (I), mimics NC (M‐nc) and inhibitor NC (I‐nc) of miR‐129 or controls (NC) into NCI‐H929 and U266 cell lines. Real‐time PCR showed a decrease or increase of miRNA in the cell lines, confirming the efficiency of transfection (Figure 2A). As shown by CCK‐8 and colony formation assays, miR‐129 overexpression was associated with a lower cell proliferation rate **(**Figure 2B‐C**)**. In addition, flow cytometry showed that the apoptosis rate in cells transfected with miR‐129 mimics was higher than that in NC **(**Figure 2D). Western blot analysis showed that overexpression of miR‐129 elevated the level of Bax protein by about 35% and lowered the level of Bcl‐2 protein by about 30% (Figure 2E). These observations were suggestive of miR‐129 as a tumour suppressor in myeloma.

### 3.3 miR‐129 overexpression suppresses tumour growth in mice

**Figure 3:**
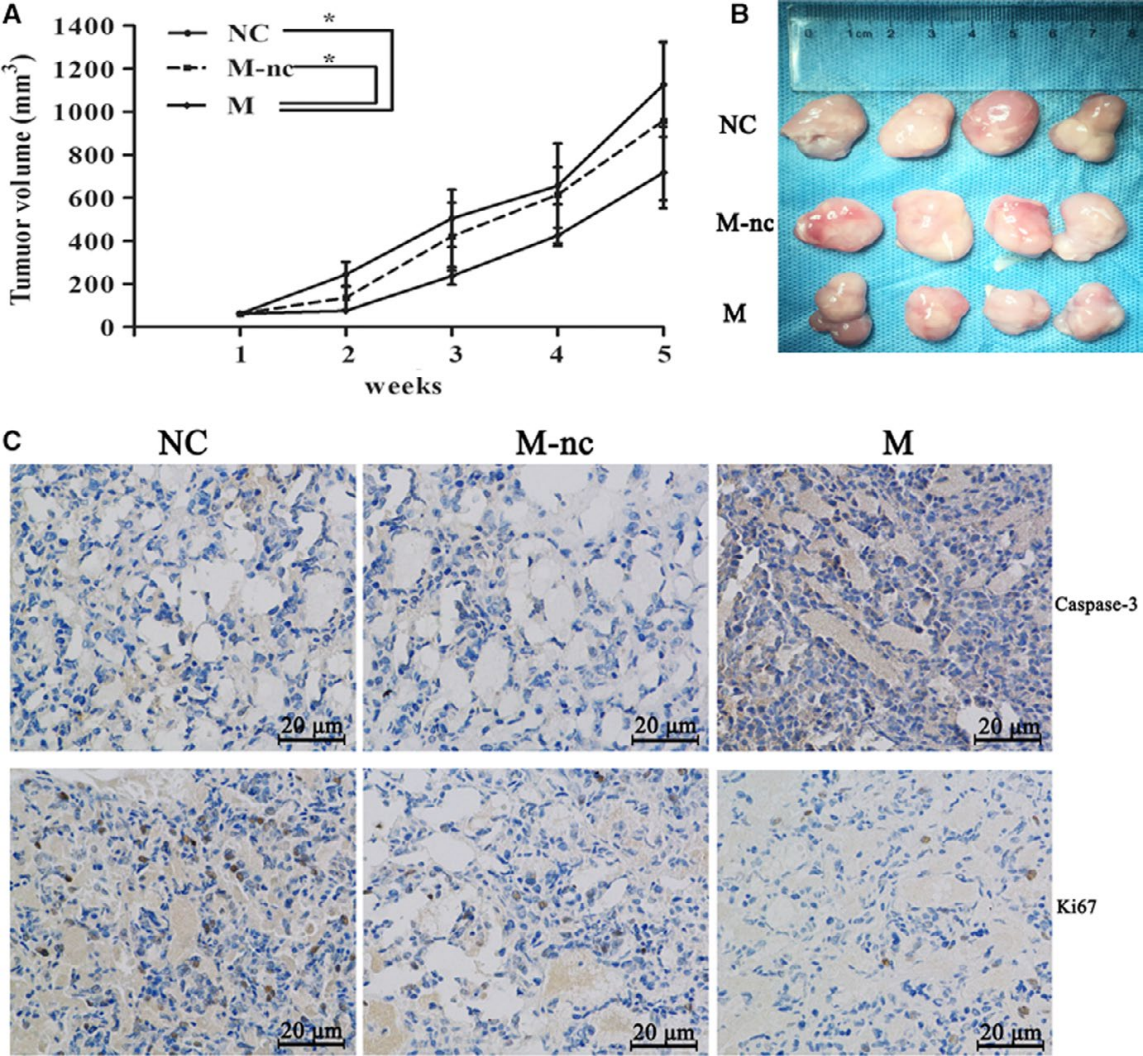
MiR‐129 overexpression suppresses tumour growth in vivo*.* A, Analysis of tumour volume curves of mice using miR‐129 mimics (M), miR‐129 mimics NC (M‐nc) or NC. B, Showing the tumours. C, Immunohistochemical staining of Ki‐67 and caspase‐3 to study proliferation and apoptosis (200×). The results could be reproduced in 5 independent experiments, mean ± SD, *P < .05

To explore the association between miR‐129 and the growth of MM in vivo, U266 cells transfected with miR‐129 mimics (M), miR‐129 mimics NC (M‐nc) or controls (NC) were injected into nude mice to established xenograft tumour model. The growth curve for tumours showed that miR‐129 overexpression inhibited tumour growth (Figure 3A). The tumour volumes in the M group were reduced significantly as compared with those in M‐nc and NC groups (*P* = .029, *P* = .039) (Figure 3B). Then, immunohistochemistry for Ki67 and caspase‐3 was performed in the xenografted tissues, showing that a higher level of miR‐129 was associated with a lower amount of caspase‐3/Ki67‐positive cells (Figure 3C). To summarize, an inhibitory effect on tumour growth was observed in the nude mouse xenograft model following miR‐129 overexpression.

### 3.4 MAP3K7 directly interacts with miR‐129

**Figure 4:**
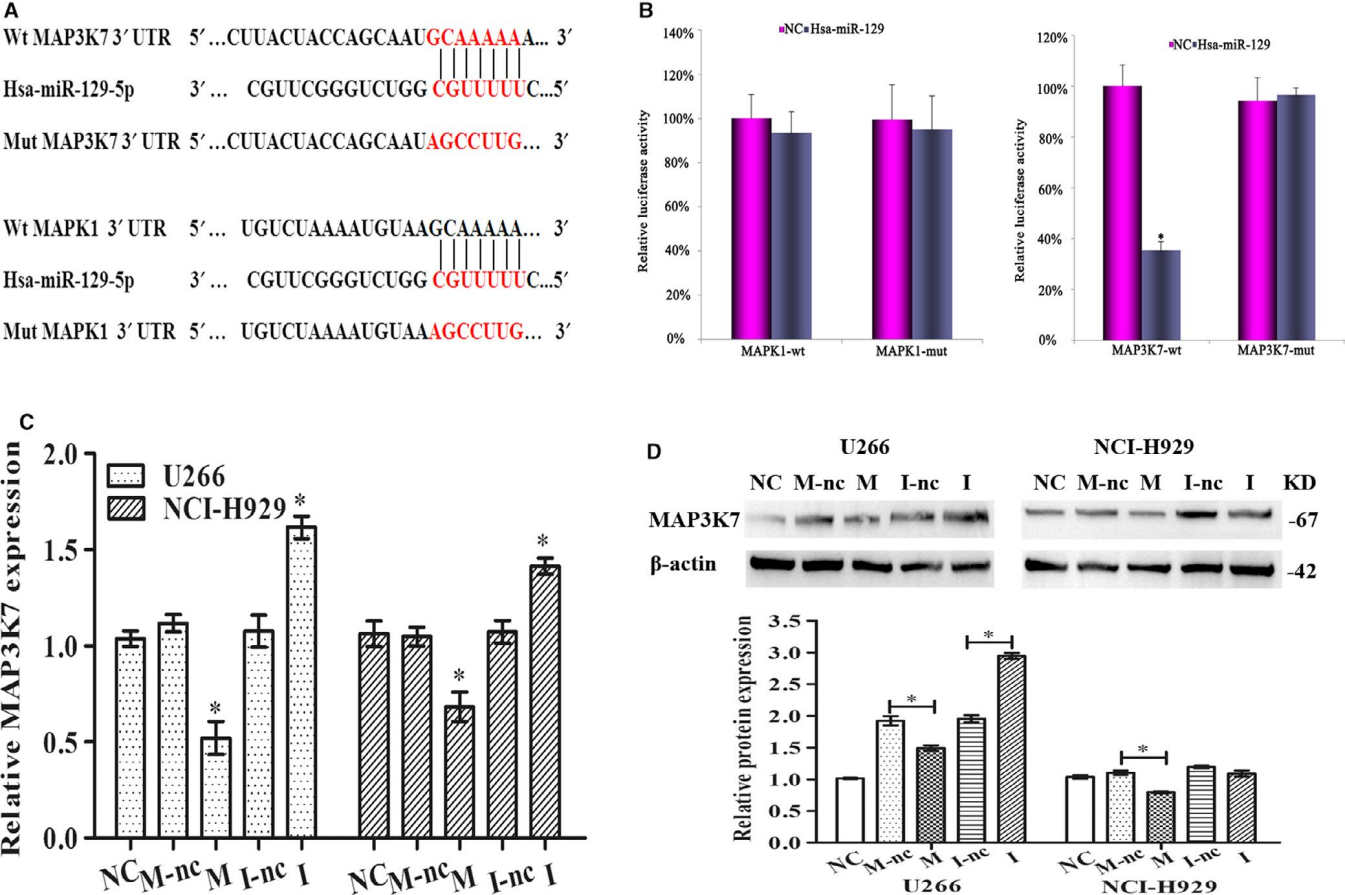
MAP3K7 directly interacts with miR‐129. A, The binding site of MAP3K7 and MAPK1 with miR‐129. The region of binding was mutated in the 3’‐UTR of the latter that associates with the seed region of the former. B, Cotransfection of MAP3K7‐wt and MAPK1‐wt and MAP3K7‐mut and MAPK1‐mut in HEK‐293T with NC and miR‐129 mimic. The activity of reporter was subjected to normalization in the control for relative expression. MAP3K7 level at transcriptional and translational levels by real‐time PCR (C) and Western blot analyses (D) with transfection of NCI‐H929 and U266 by NC, mimics NC (M‐nc), mimics (M), inhibitor NC (I‐nc) and inhibitor (I) for miR‐129; GAPDH was control. Triplicate verification of results was achieved, mean ± SD, *P < .05

Several bioinformatics databases and software systems concerning TargetScan, microRNA.org and DIANA‐miT were utilized to scan for the targets of miR‐129. MAP3K7 and MAPK1 were thought to be the targets due to their match in the miRNA “seed sequence” (Figure 4A). Then, enzyme reporter assay was applied to confirm their interaction. It was found that the enzyme activity was inhibited significantly at a higher amount of miR‐129 when the 3′ UTR of MAP3K7 was wild‐type as against the mutated sequence (*P* < .05) (Figure 4B). Additionally, MAP3K7 was inhibited markedly both at the transcriptional and translational levels by miR‐129 overexpression in NCI‐H929 and U266 lines. An opposite tendency was observed with higher RNA and protein levels of MAP3K7 when miR‐129 was suppressed (*P* < .05) (Figure 4C‐D). The observations were suggestive of modulation of MAP3K7 by miR‐129.

### 3.5 The NF‐κB pathway is involved in MAP3K7 and miR‐129 functions in MM

**Figure 5:**
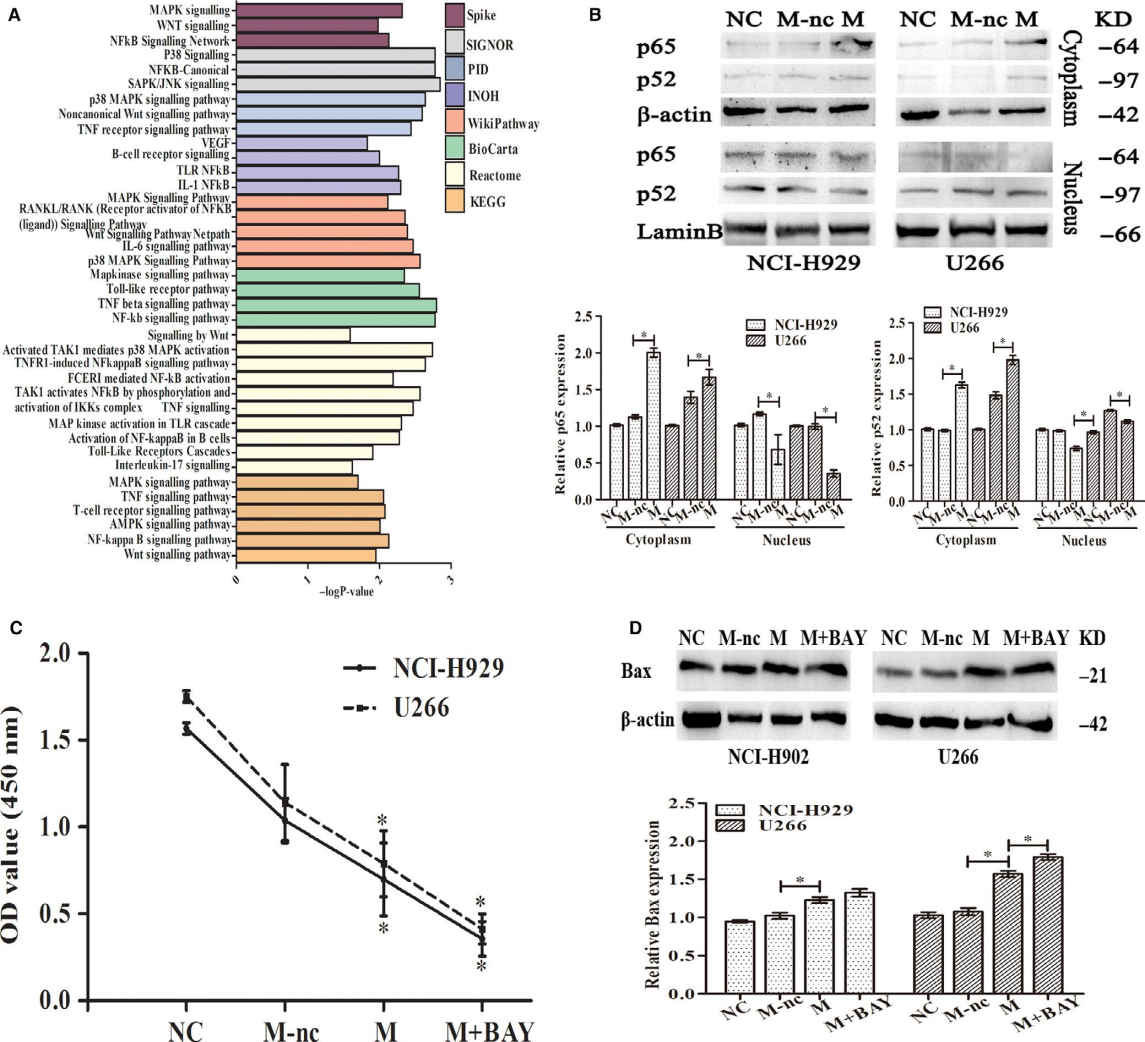
miR‐129‐mediated MAP3K7 induces a biological function on MM cells via the NF‐κB signalling pathway. A, PathDIP was used to analyse the significantly enriched pathways of MAP3K7 gene. B, Transfection of NCI‐H929 and U266 with NC, mimics NC (M‐nc), mimics (M) of miR‐129 for 48 h. Antibodies to p65/p52 were used for Western blot. Post‐blot stripping was done and studied with lamin B and β‐actin for the nucleus and cytoplasm, respectively, to ascertain equal loading. C, Transfecting NCI‐H929 and U266 cells with NC, mimics NC (M‐nc), mimics (M) of miR‐129 for 48 h and BAY‐11‐7082 treated for 24 h. CCK‐8 to assess division. D, Western blot for proteins involved in apoptosis. Triplicate assays were conducted, mean ± SD, *P < .05

To examine the mechanisms of the expressed biological function, we analysed signalling pathways through pathDIP to find the significantly enriched pathways of MAP3K7 gene. As shown in Figure 5A, several pathways were associated with MAP3K7, including the “NF‐κB pathway” and “MAPK pathway,” and activated TAK1 mediated “p38 MAPK activation,” “WNT signalling” and “SAPK/JNK signalling,” suggesting that MAP3K7 played a role in activating NF‐κB by acting on IKK complex via phosphorylation, causing ubiquitination degradation of NFKB1A or IκBα, resulting in freeing NF‐κB that was translocated to the nucleus after activation.^12^ We therefore inferred that this pathway may be inhibited by miR‐129 overexpression. Western blot showed that translocation of p65 and p52 in MM cells was lowered by of miR‐129 overexpression (Figure 5B). To investigate whether NF‐κB signalling participated in the biological effects of miR‐129 observed above, overexpressed miR‐129 along with the NF‐κB inhibitor (BAY‐11‐7082) was operated simultaneously in MM cells. It was found that NF‐κB inhibitor enhanced an inhibitory effect of miR‐129 overexpression on disturbed cell proliferation and spurred apoptosis (Figure 5C‐D). To summarize, the NF‐κB pathway played a role in the function of miR‐129 mediated in MM.

### 3.6 PCAT‐1 acts as a sponge of miR‐129

**Figure 6:**
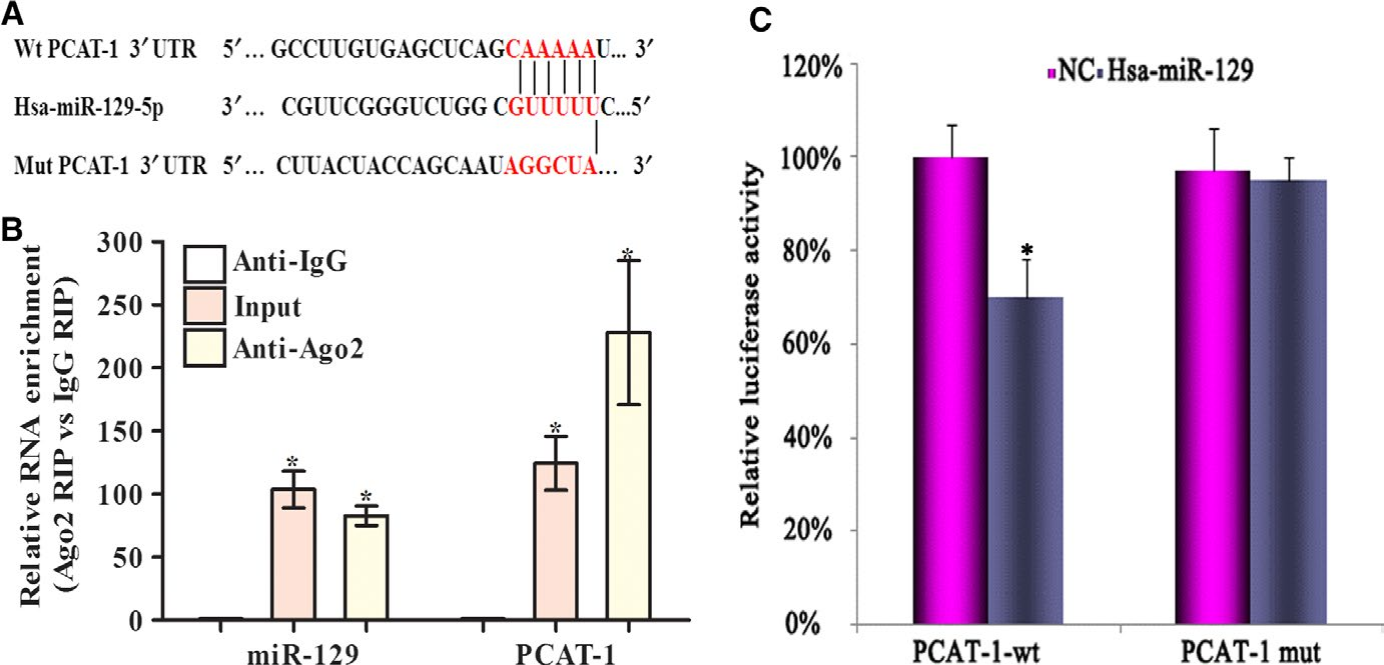
PCAT‐1 acts as a sponge of miR‐129. A, Mutation of the PCAT‐1‐miR‐129 potential region. B, RIP for Ago2 antibody on U266. The amount of PCAT‐1 and miR‐129 was measured by real‐time PCR. The levels of RNA were found to be enriched in terms of the control IgG. C, Cotransfection of PCAT‐1‐wt and PCAT‐1‐mut in HEK‐293T with miR‐129 mimics or control. The relative enzyme activity was considered post‐normalization in terms of the control. Triplicate assays were conducted, mean ± SD, *P < .05

Research has shown that most lncRNAs work as a molecular sponge to reduce the ability of miRNAs to bind with their targets and the miRNA activity at a post‐transcriptional level. According to the online software MicroInspector (http://bioinfo.uni-plovdiv.bg/microinspector/), we observed that PCAT‐1 contained predicted targeting sites of miR‐129 (Figure 6A). Knowing that Ago2 protein is a vital part of the RNA‐induced silencing complex (RISC), we conducted a RIP experiment in U266 lines using Ago2 antibody to investigate potential involvement of PCAT‐1 and miR‐129 complex. The precipitates were then checked by real‐time PCR. The Ago2 fraction contained enrichment for PCAT‐1 and miR‐129 by a factor of 224 and 83, respectively, as compared with the controls in lieu of our expectations. This was suggestive of the presence of PCAT‐1 in Ago2 RISC via sponging miR‐129 (Figure 6B). This was further confirmed by luciferase reporters with both wild‐type and mutated sites for binding miR‐129. The enzyme activity was lowered when miR‐129 mimics were transfected for PCAT‐1‐wt while that of PCAT‐1‐mut and vector controls showed no change (Figure 6C). All these results suggest that PCAT‐1 played a role in down‐regulating miR‐129.

### 3.7 PCAT‐1 is a modulator in miR‐129/MAP3K7 regulatory network

**Figure 7:**
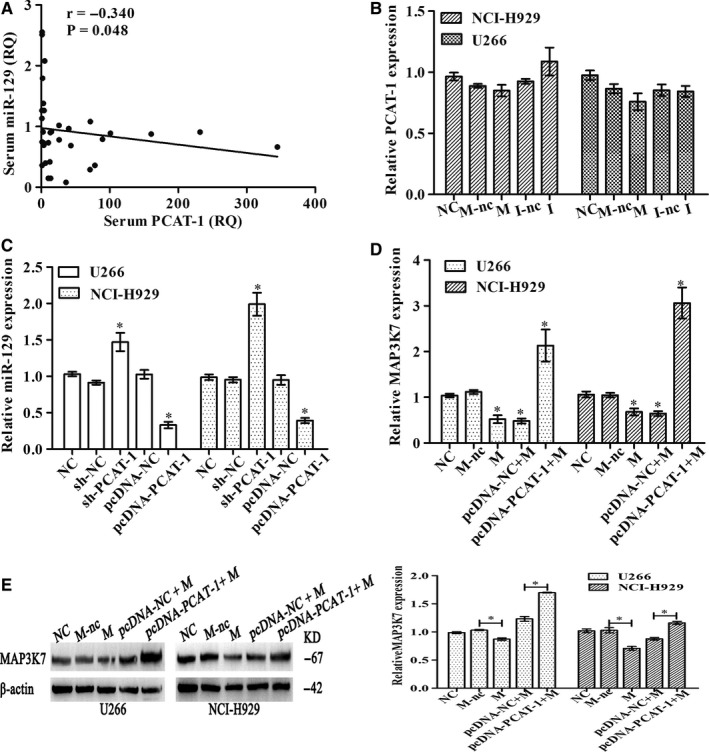
Verification of the association between PCAT‐1 and MAP3K7. A, PCAT‐1 and miR‐129 correlation from sera of 34 MM patients. B, NCI‐H929 and U266 were transfected with NC, mimics NC (M‐nc), mimics (M), inhibitor NC (I‐nc) and inhibitor (I) for miR‐129 for 48 h. PCAT‐1 was assayed by real‐time PCR. C, Detection of miR‐129 after 48‐h transfection of NCI‐H929 and U266 with NC, sh‐NC, sh‐PCAT‐1, pcDNA‐NC and pcDNA‐PCAT‐1. MAP3K7 was detected at transcriptional and translational levels by real‐time PCR (D) and Western blot (E) following treatment of NC, sh‐NC, sh‐PCAT‐1, pcDNA‐NC and pcDNA‐PCAT‐1. Triplicate verification of the observations was achieved, mean ± SD, *P < .05

The relative expression between miR‐129 and PCAT‐1 expressions was found to be inversely correlated in the serum samples of MM patients **(**Figure 7A**)**. Transfection of miR‐129 mimics into U266 and NCI‐H929 lines showed no decrease in PCAT‐1 expression as compared with mimic NC (Figure 7B). However, PCAT‐1 knockdown led to a striking rise in miR‐129 in comparison with the control, while overexpression of PCAT‐1 lowered miR‐129 (Figure 7C). Furthermore, the role of PCAT‐1 in modulating miR‐129 via MAP3K7 was assessed by transfection of sh‐PCAT‐1 and miR‐129 mimic followed by analysis of the MAP3K7 RNA and protein. Decreased MAP3K7 was perceived by the miR‐129 mimics both at the transcriptional and translational levels, but pcDNA‐PCAT‐1 combined with miR‐129 mimics restored the reduction of MAP3K7 expression significantly (Figure 7D‐E). These results of experiments suggested that PCAT‐1 sponges miR‐129 to regulate MAP3K7 in the myeloma.

### 3.8 The inhibitory effect of miR‐129 is reversed by PCAT‐1

**Figure 8:**
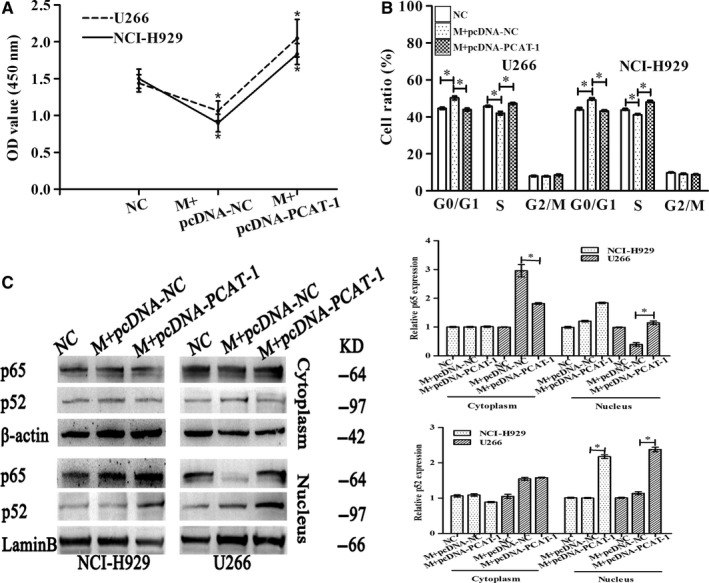
miR‐129 manifestations (cell growth/NF‐κB) are reversed by PCAT‐1. Transfection of NCI‐H929 and U266 with NC, mimics (M)+pcDNA‐NC, mimics (M)+pcDNA‐PCAT‐1 for miR‐129 for 48 h. A, The proliferation of MM cells was detected by CCK‐8. B, The cell cycle was detected by flow cytometry. C, The expression of p65 and p52 proteins was detected by Western blot analysis. Triplicate assays were conducted, mean ± SD, *P < .05

As miR‐129 expression could be modulated by PCAT‐1, an association in terms of functional phenotypes was also examined. Cotransfection with pcDNA‐PCAT‐1 and miR‐129 mimic in U266 and NCI‐H929 cells augmented the viability of cells and restored their growth as compared with mimics NC/pcDNA‐NC (Figure 8A). Cotransfection increased the proportion of cells at the S phase vs G0/G1 phase, and there was a higher proportion of miR‐129 mimic‐transfected cells in G0/G1 (Figure 8B). Nuclear translocation of p65 and p52 protein was sharply increased in miR‐129 mimics and PCAT‐1 cotransfected groups as compared with NC or miR‐129 mimics and pcDNA‐NC co‐treatment groups in U266 and NCI‐H929 cells (Figure 8C). The tumour suppressor functions of miR‐129 on cell growth and signalling pathway were reversed by PCAT‐1.

## 4 DISCUSSION

The involvement of miRNAs in several malignancies including MM suggests their potential as biomarkers and therapeutic targets.^13^, ^14^, ^15^, ^16^ Yet, the miRNAs involved in MM have not been clearly defined. In this study, we for the first time demonstrated that the miR‐129 expression was decreased significantly in newly diagnosed MM patients as compared with that in the healthy control. Depending on the monoclonal immunoglobulin secreted by MM cells, MM can be classified into the following several types, such as IgG, IgD, IgA, IgE and IgM. We also found that there was an association between the expression level of serum miR‐129 and different MM isotypes in these MM patients, which might help to distinguish different immune types of multiple myeloma, suggesting miR‐129 and its upstream regulator played a regulatory role in MM.

Previous studies^17^ reported that down‐regulated expression of miR‐129 in the tissue samples and cell lines of papillary thyroid cancer inhibited tumour cell growth by targeting KLK7. Besides, miR‐129 was reported to suppress cell division and invasion in gastric cancer via regulating ADAM9.^18^ However, the elevated expression of miR‐129 enhanced the ability of CRC cells to proliferate and migrate.^10^ With respect to MM, the roles and inner mechanisms of miR‐129 have not yet been elaborated. This study revealed a negative impact of higher levels of miR‐129 on proliferation and cell cycle and a positive impact on apoptosis of myeloma cells, and these effects could be reversed by knocking down miR‐129. Moreover, in vivo assay showed that miR‐129 overexpression weakened tumour growth in a mouse model. The results were suggestive of the tumour‐suppressive effect of miR‐129.

MAP3K7, referred to as TGF‐β‐activated kinase‐1 (TAK1), was identified to be targeted by miR‐129. Under normal physiological conditions, MAP3K7 comes under the aegis of mitogen‐activated protein kinase (MAP3K) that undergoes quick response to TGF‐β.^19^ This protein is also found to regulate several pathways, such as JNK and NF‐κB pathway, that are commonly reported to participate in carcinogenesis.^20^ MAP3K7 inhibition is associated with the tumour survival rate. Hence, it is of interest to explore its role in therapeutic interventions.^21^ It was found in our luciferase reporter assay that there was a binding site between MAP3K7 and miR‐129, and an inverse correlation between MAP3K7 and miR‐129 in the serum samples of MM patients, which discloses the role of MAP3K7 as a direct target of miR‐129.

Earlier work 22, 23 showed inhibition of MAP3K7 compromised tumour growth and metastasis in breast cancer and stalled the progression of leukaemia in an AML xenograft model.^24^ In addition, MAP3K7 participated in the evolution of inflammation, tumour‐associated immunoreactivity and apoptosis inhibition via NF‐κB activation.^25^ In fact, NF‐κB was elevated in many malignancies (including MM) where its activation was associated with fast division, tumour growth, diminished apoptosis and wide drug resistance.^26^ This study showed that overexpression of miR‐129 markedly lowered the enrichment of p65 and p52 protein in the nucleus. To summarize, the involvement of MAP3K7 in controlling the NF‐κB circuit, which was further regulated by miR‐129, compromised carcinogenic manifestations.

The diminished expression of miR‐129 in MM cell lines was further explored. MiR‐129 was reported previously to be regulated by lncRNAs.^27^, ^28^ In this study, we for the first time demonstrated a direct link between PCAT‐1 and miR‐129 in MM and that the PCAT‐1 level in MM‐derived PCs was significantly higher than that in the control and negatively associated with the level of serum miR‐129. Altering the levels of PCAT‐1 was pursued to explore its functions. PCAT‐1 overexpression was found to augment cell growth and division, while PCAT‐1 inhibition produced an opposite effect, suggesting that PCAT‐1 worked as an oncogene o in MM.

Previous studies^29^, ^30^ have reported a mode of lncRNA as ceRNA to silence miRNAs. RIP and luciferase reporter assay explored potential sites of PCAT‐1‐associated miR‐129 and found a direct association between miR‐129 and PCAT‐1. It was also found that PCAT‐1 knockdown or miR‐129 mimics alone reduced the expression of MAP3K7, while combination of pDNA‐PCAT‐1 with miR‐129 mimics restored the reduction of MAP3K7 expression significantly.

To summarize, we demonstrated that PCAT‐1 worked a ceRNA to regulate MAP3K7 and negatively modulate miR‐129 in MM.

## 5 CONCLUSION

Diminished expression in both MM‐derived PCs and serum samples suggested that miR‐129 played a tumour‐suppressive role in MM. Augmenting the miR‐129 expression adversely affected various phenotypes of carcinogenesis, cell growth and apoptosis. In addition, accumulation of lncRNA PCAT‐1 induced miR‐129 loss and promoted cell division and cell cycle evolution, and diminished apoptosis through the MAP3K7/NF‐κB pathway. A comprehensive understanding about the above genes would help uncover newer approaches to address MM. It is expected that several miRNAs can be regulated by PCAT‐1 with each miRNA controlling specific or several signalling molecules and targets to thus, which overall affects MM progression. However, more experimental and animal studies are required to identify more functional lncRNAs associated with MM for the sake of developing effective strategies for the clinical treatment of MM.

## CONFLICT OF INTEREST

None.

## AUTHOR CONTRIBUTIONS

XJS participated in cell culture and drafted the manuscript. QY and QQY carried out the immunoassays. HC and XDW carried out cell functional tests and PCR assay. SQJ and SK conceived, co‐ordinated the study and analysed data. All authors reviewed and edited the manuscript and approved the final version.

## ETHICS APPROVAL AND CONSENT TO PARTICIPATE

The local ethics committee issued approval of the protocols involved with requisite consent from the patients. The animal experiments were applied according to the guidelines of the Animal Care Committee of Nantong University.
