# Corrigendum

**DOI:** 10.1111/jcmm.17425

**Published:** 2022-07-06

In Xianjuan Shen et al.,^1^ the flow cytometric analysis in Figure 2D and the immunohistochemical staining in Figure 3C are incorrect. The correct figures are shown below. The authors confirm that all results and conclusions of this article remain unchanged.

**FIGURE 2:**
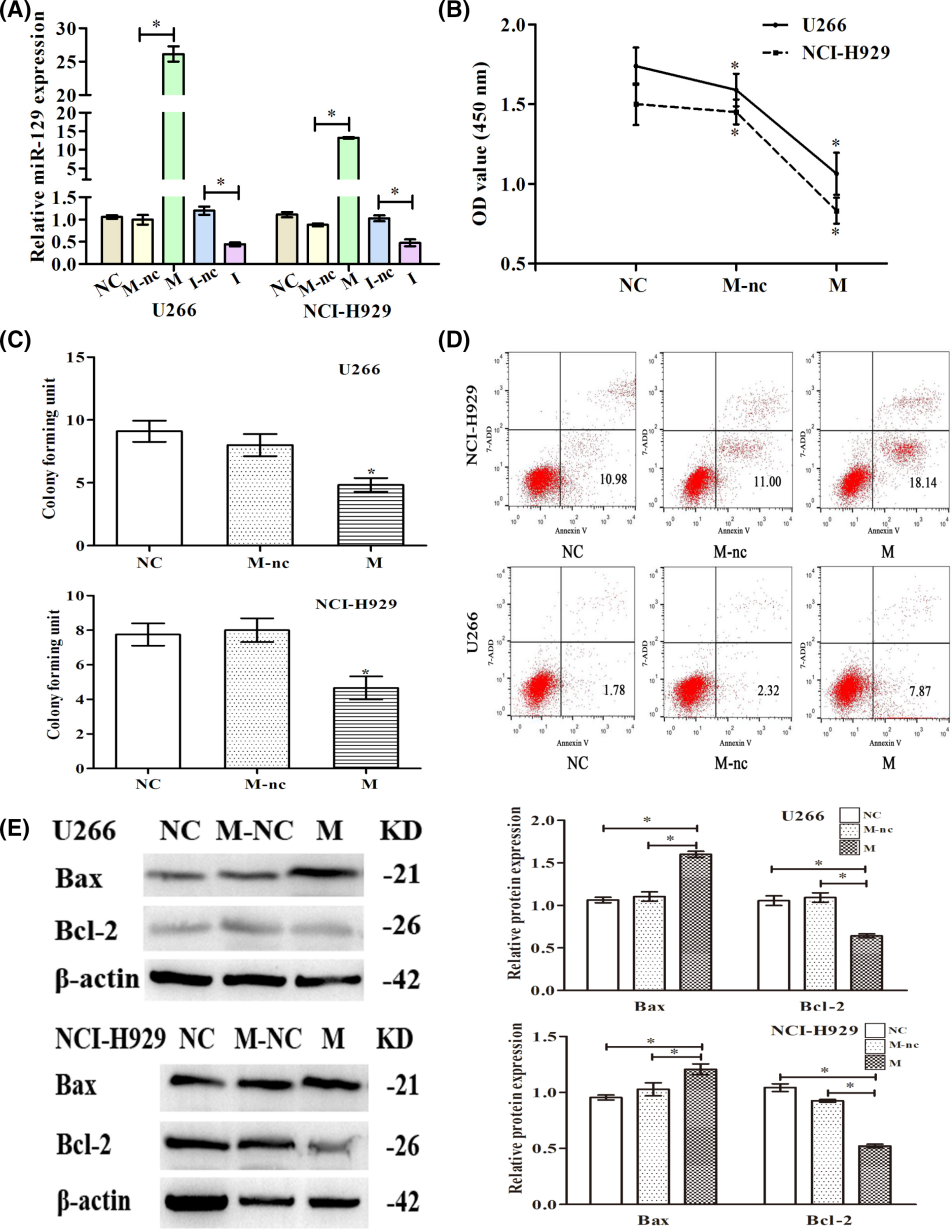
miR‐129 arrests the cell cycle, reduces the ability of cells to proliferate and induces apoptosis. Transfection of NCI‐H929 and U266 with miR‐129‐mimics (M), miR‐129‐mimics‐NC (M‐nc), miR‐129‐inhibitor (I), miR‐129‐inhibitor‐NC (I‐nc) or controls (NC). (A) Detection of the relative expression of miR‐129 after transfection by real‐time PCR. (B) Detection of cell proliferation by CCK‐8. Cell proliferation inhibition rates of U266 and NCI‐H929 cells in the M group were about 35% and 42%. (C) The number of cloned cells as shown by soft agar colony formation assay. (D) The number of apoptotic cells by flow cytometric analysis. Five independent experiments were conducted. M group owned higher apoptosis rate than the M‐nc and NC groups in U266 (*p* = 0.006, *p* = 0.007) and NCI‐H929 cells (*p* = 0.011, *p* = 0.006). (E) Western blot for expression of Bcl‐2/Bax (apoptotic proteins). The results could be reproduced in three independent experiments, mean ± SD, *p < 0.05

**FIGURE 3:**
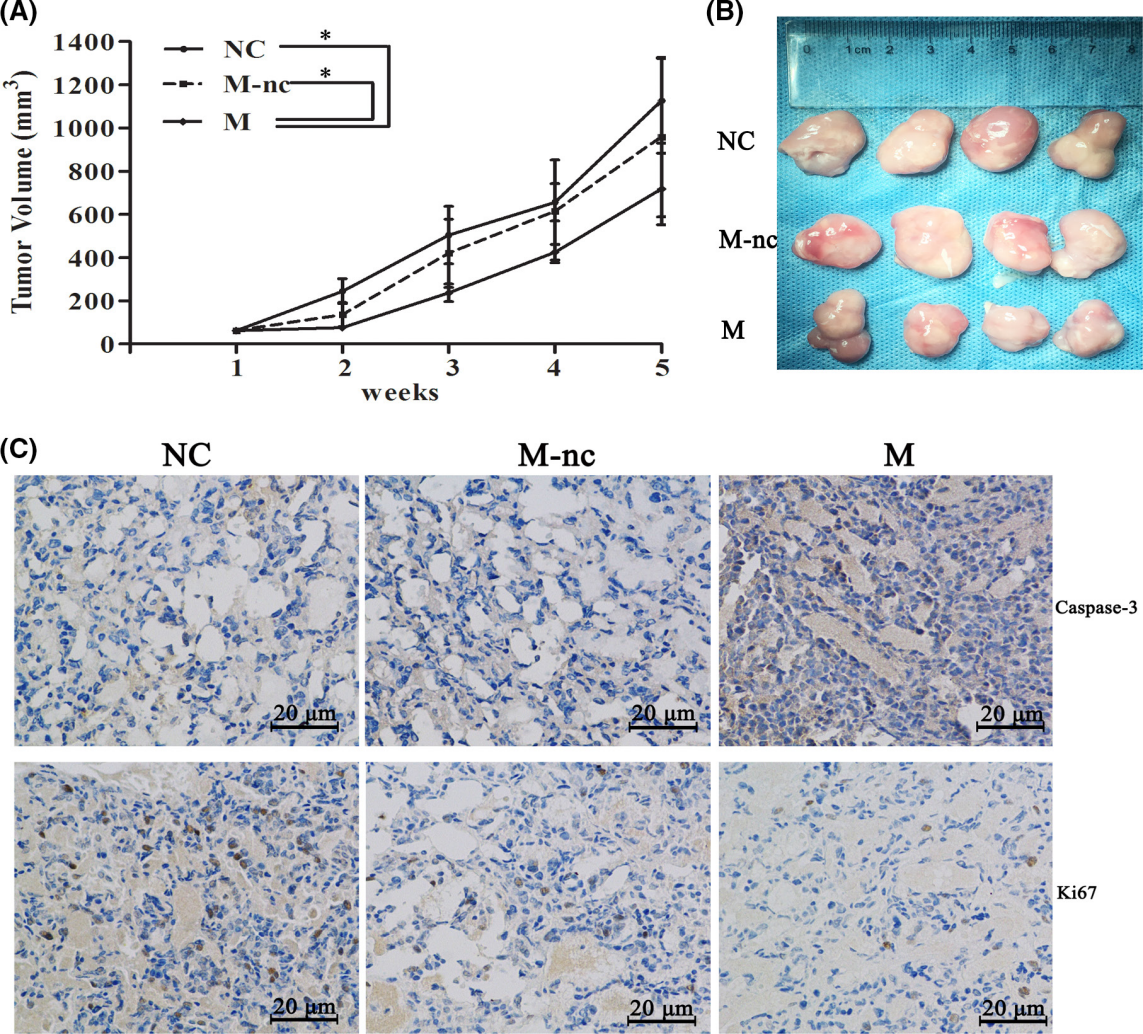
MiR‐129 overexpression suppresses tumour growth in vivo. (A) Analysis of tumour volume curves of mice using miR‐129 mimics (M), miR‐129‐mimics‐NC (M‐nc) or NC. (B) Showing the tumours. (C) Immunohistochemical staining of Ki‐67 and caspase‐3 to study proliferation and apoptosis (200×). The results could be reproduced in five independent experiments, mean ± SD, *p < 0.05

The description of Figure 3C in the published manuscript was corrected as follows: Then, immunohistochemistry for Ki67 and caspase‐3 was performed in the xenografted tissues, showing that a higher level of miR‐129 was associated with a higher amount of caspase‐3 and a lower amount of Ki67‐positive cells, respectively (Figure 3C).
